# Cardiac amyloidosis and surgery: What do we know about rare diseases?

**DOI:** 10.1111/jocs.15630

**Published:** 2021-05-16

**Authors:** Carlos A. Mestres, Mathias Van Hemelrijck

**Affiliations:** ^1^ Clinic of Cardiac Surgery University Hospital Zürich Zürich Switzerland; ^2^ Department of Cardiothoracic Surgery The University of the Free State Bloemfontein South Africa

**Keywords:** cardiac amyloidosis, cardiac surgery, rare disease

Rare diseases are serious, chronic, and potentially lethal. The European Union (EU) definition of a rare disease is one that affects fewer than 5 in 10 000 people.^1^ In the EU, these rare diseases are estimated to affect up to 8% of the roughly 500 million population.^2^ In the United States, a rare disease is defined as a condition affecting fewer than 200 000 people (https://rarediseases.info.nih.gov/diseases). This is a definition created by Congress in the Orphan Drug Act of 1983.^3^ Therefore, the estimates for the United States are that 25–30 million people are affected by a rare disease. There are more than 6000 rare diseases and 80% are genetic disorders diagnosed during childhood. Despite all community efforts, there is still a lack of a universal definition of rare diseases. This was addressed a few years back by the International Society for Pharmacoeconomic and Outcomes Research (ISPOR).^4^

Amyloidosis is a rare disorder classified in different types. It is registered in the National Organization for Rare Disorders (NORD) database (https://rarediseases.org), where a report can be found addressing the fundamentals for patients, relatives, and clinicians. Although classified as a rare disease, it seems that amyloidosis is increasingly being recognized as a cause of heart failure.^5^ The European Society of Cardiology has promptly recognized this in a recent position paper published by its Working Group on Myocardial and Pericardial Disease.^6^ Its potential role in heart failure with preserved ejection fraction and eventual therapeutic approach have recently been highlighted in some reviews.^7^, ^8^

Having said that, Smith et al.^9^ from London present in this issue of the journal, an interesting review proposal with regards to cardiac amyloidosis in non‐transplant cardiac surgery. As briefly stated by the authors, heart transplantation is becoming the ultimate therapeutic tool in light‐chain amyloidosis and transthyretin amyloidosis, with some interesting short‐term outcomes reported in some contributions, like the recent one from Vaidya et al.^10^ These authors report an overall 3‐year survival of 81.6% in a series of 51 patients. However, this is not, as described in their title, the focus of the authors.

With the assumption that heart transplantation works acceptably in selected patients, an important statement in this contribution by Smith et al.^9^ is that it is difficult to find a strong justification to recommend a cardiac surgical intervention in patients with cardiac amyloidosis. There are reasons to understand that these patients, regardless of the clinical problem eventually requiring cardiac surgery, namely aortic stenosis, mitral disease, or coronary artery disease amenable for surgical revascularization, have a clear profile for intra‐ and postoperative complications in the form of low‐output syndrome entailing significant mortality. The pathophysiological issues surrounding cardiac amyloidosis, meaning a restrictive physiologic with diastolic dysfunction and substance myocardial deposition configure a suboptimal pattern for extended survival.

There are still a number of issues to discuss, like ethnicity,^11^ familial association, the prognosis on the long‐term or, even, if the pandemic has brought additional burden to these patients.^12^ However, it is not to be forgotten that this is technically a rare disease and collective experience, especially in the surgical field, is still scanty. In their review, Smith et al.^9^ discuss a number of small‐sized studies and mortality. Most of these studies do not report the actual cause of death. As the authors discuss the potential confounders of all these studies, not knowing the actual cause of death or, as usual, not having postmortem examinations, all goes around confounders and speculation.

The readership should not incorporate confusing information. As per some types of reports in the literature, it may seem that each and every cardiac disease is amyloid‐related. It is also clear that specifically dedicated centers, like the NHS National Amyloidosis Centre in London Amyloidosis Center, Boston University School of Medicine, and others, may have much more information as they can collect more cases and discuss cardiac amyloid.

After their review with a focus on diagnosis, surgical risk, and areas of uncertainty that require further research, some take‐home messages from Smith et al.^9^ are that cardiac amyloidosis, per se, has an intrinsic poor prognosis, that surgical treatment of diseases that may have an amyloid component like aortic stenosis need an extremely careful and accurate individualized assessment and that, currently, heart transplantation has to be considered in specific subgroups. For the time being, the indications for non‐transplant cardiac surgery seem to be restrictive. Do not forget, as stated, that rare diseases were also called “orphan diseases,” and largely ignored due to poor economic potential and were thus said to be “orphaned.”^12^ Some more time may then be needed to understand what the role of surgery may be.
